# H3K27 acetylation and gene expression analysis reveals differences in placental chromatin activity in fetal growth restriction

**DOI:** 10.1186/s13148-018-0508-x

**Published:** 2018-06-26

**Authors:** N. D. Paauw, A. T. Lely, J. A. Joles, A. Franx, P. G. Nikkels, M. Mokry, B. B. van Rijn

**Affiliations:** 1 Department of Obstetrics, Wilhelmina Children’s Hospital Birth Center, University Medical Center Utrecht, Utrecht, the Netherlands; 2 Department of Nephrology and Hypertension, University Medical Center Utrecht, Utrecht, the Netherlands; 3 Department of Pathology, University Medical Center Utrecht, Utrecht, the Netherlands; 4 Division of Pediatrics, University Medical Center Utrecht, Utrecht, the Netherlands; 5 Academic Unit of Human Development and Health, University of Southampton, Southampton, UK; 6 Division Woman and Baby, University Medical Center Utrecht, Postbus 85090, 3508 AB Utrecht, the Netherlands

**Keywords:** ChIP-seq, Growth restriction, H3K27ac, Epigenetics, Histone acetylation, Placenta, Placental pathology, RNA-seq

## Abstract

**Background:**

Posttranslational modification of histone tails such as histone 3 lysine 27 acetylation (H3K27ac) is tightly coupled to epigenetic regulation of gene expression. To explore whether this is involved in placenta pathology, we probed genome-wide H3K27ac occupancy by chromatin immunoprecipitation sequencing (ChIP-seq) in healthy placentas and placentas from pathological pregnancies with fetal growth restriction (FGR). Furthermore, we related specific acetylation profiles of FGR placentas to gene expression changes.

**Results:**

Analysis of H3K27ac occupancy in FGR compared to healthy placentas showed 970 differentially acetylated regions distributed throughout the genome. Principal component analysis and hierarchical clustering revealed complete segregation of the FGR and control group. Next, we identified 569 upregulated genes and 521 downregulated genes in FGR placentas by RNA sequencing. Differential gene transcription largely corresponded to expected direction based on H3K27ac status. Pathway analysis on upregulated transcripts originating from hyperacetylated sites revealed genes related to the HIF-1-alpha transcription factor network and several other genes with known involvement in placental pathology (LEP, FLT1, HK2, ENG, FOS). Downregulated transcripts in the vicinity of hypoacetylated sites were related to the immune system and growth hormone receptor signaling. Additionally, we found enrichment of 141 transcription factor binding motifs within differentially acetylated regions. Of the corresponding transcription factors, four were upregulated, SP1, ARNT2, HEY2, and VDR, and two downregulated, FOSL and NR4A1.

**Conclusion:**

We demonstrate a key role for genome-wide alterations in H3K27ac in FGR placentas corresponding with changes in transcription profiles of regions relevant to placental function. Future studies on the role of H3K27ac in FGR and placental-fetal development may help to identify novel targets for therapy of this currently incurable disease.

**Electronic supplementary material:**

The online version of this article (10.1186/s13148-018-0508-x) contains supplementary material, which is available to authorized users.

## Background

The dynamics of histone 3 lysine 27 acetylation (H3K27ac) in DNA regulatory regions is one of the components playing a fundamental role in the precise timing and level of gene transcription [1, 2]. Consequently, aberrant H3K27ac has been suggested to be involved in disease pathology by eliciting pathological gene expression programs [3, 4]. H3K27ac marks both active promoters and distal enhancers, the most important and best understood regulatory domains. To study involvement of this regulatory level/layer in placental pathology, we mapped H3K27ac occupancy in healthy placentas and placentas from pregnancies with fetal growth restriction (FGR).

FGR, a condition in which the fetus is unable to achieve its full growth potential through inadequate supply of nutrients and growth factors, occurs in approximately 5% of pregnancies [5]. FGR imposes a major risk of perinatal morbidity and mortality [6] and programs the health of the fetus throughout life, by being associated with a future risk of type 2 diabetes and cardiovascular and renal disease [7, 8]. At the histopathological level, FGR placenta exhibit signs of disrupted placental development characterized by increased infarction area, increased syncytial knotting, inflammation, and impaired trophoblast invasion into the spiral arteries of the uterus due to inappropriate maternal-fetal immune interaction [9–11].

Previous studies of placentas of FGR pregnancies have reported differences in gene transcription across many regions across the genome. Pathways associated with altered placental gene expression in FGR include angiogenesis, immune modulation, energy production, and growth signaling [12–15]. Most adaptive responses of the placenta, e.g., to support restricted fetal growth, are thought to result from changes in epigenetic regulation [16–18]. Recently, DNA methylation has been mapped in the human placenta [19] and a number of studies have suggested differences in genome-wide methylation profiles in FGR placentas [20–22]. We assume that disruptions in other epigenetic systems (e.g., histone modifications and other posttranslational chromatin modifications) regulating placenta gene expression are also likely to be involved [23, 24].

In this study, we mapped differential H3K27ac profiles in DNA regulatory regions in relation to disrupted development of the placenta, by exploring the presence of H3K27ac using chromatin immunoprecipitation sequencing (ChIP-seq) in FGR and control placentas. Next, we performed RNA-seq to examine whether differences in H3K27ac also reflects gene expression levels. With this approach, we identified previously unstudied alterations in promoter and enhancer activity related to placenta pathology.

## Results

### Detection of regions with differential H3K27ac occupancy

**Fig. 1:**
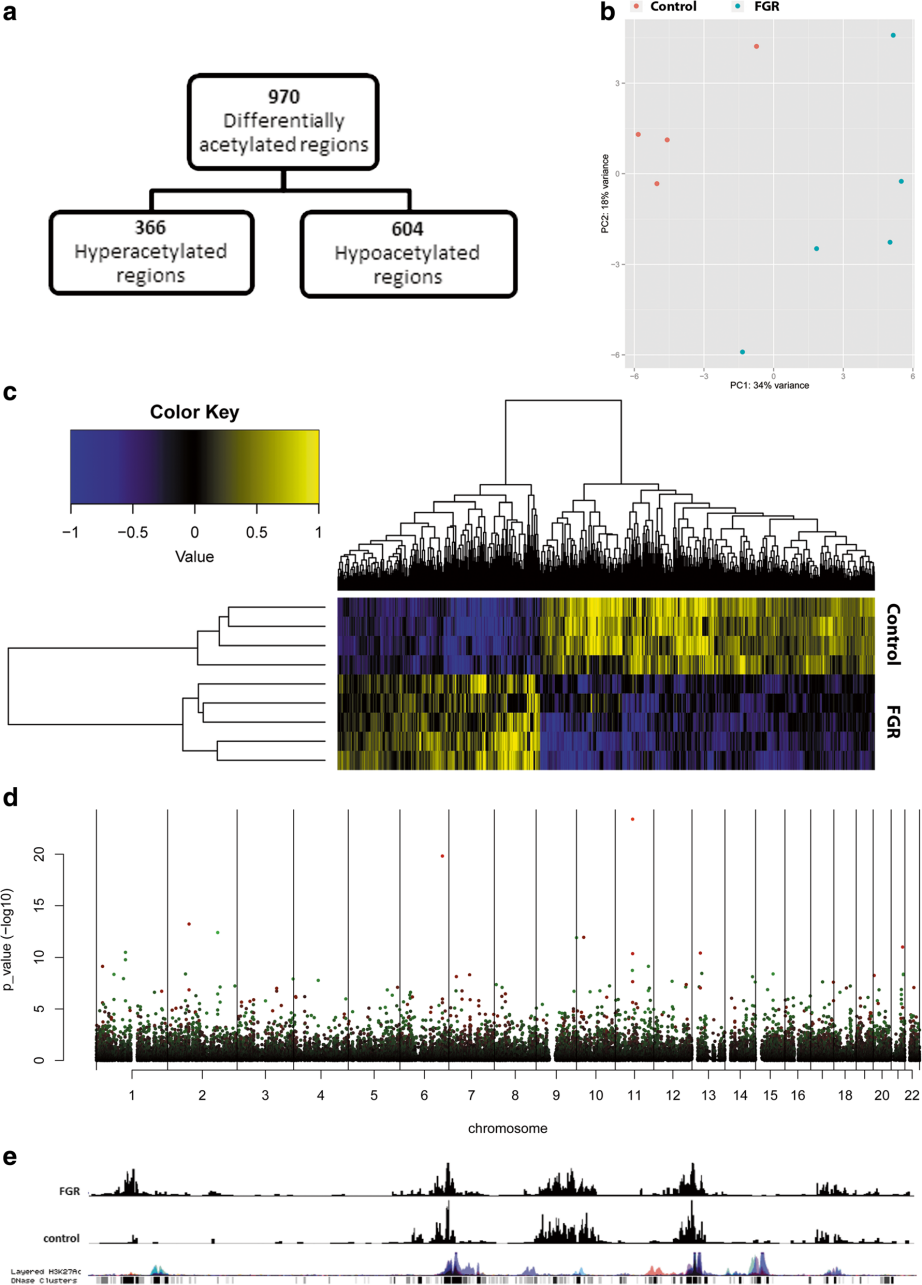
Detection of H3K27ac occupancy in placentas of FGR and controls by CHIP-seq. **a** Flowchart of differentially acetylated region in FGR vs. control placentas (adjusted *p* < 0.05). **b** Heatmap of differentially acetylated regions between FGR and controls. **c** PCA clustering of the 500 most variable acetylated regions based on H3K27ac ChIP-seq signal between FGR and controls. **d** Manhattan plot depicting distribution of differentially H3K27 acetylated regions in FGR vs. controls: non-significant regions (black), hyperacetylated regions (green), and hypoacetylated regions (red). **e** Selected peaks from Chr11 showing hypoacetylation in FGR vs. controls with ENCODE as reference

First, we performed genome-wide analysis of H3K27ac by ChIP-seq on placental tissue from control (*n* = 4) and FGR pregnancies (*n* = 5) and identified 30,288 H3K27ac peaks that were present in at least two independent samples. Of these, 970 regions showed differential H3K27ac levels in FGR compared to controls with 366 being hyperacetylated and 604 regions being hypoacetylated in FGR (Fig. 1a, full list supplied in Additional file 1: Table S1). Based on the differentially acetylated regions, the FGR and healthy placentas could be clearly segregated using both supervised and unsupervised analysis (Fig. 1b–c, Additional file 2) These findings indicate clear distinction between the two groups and suggest a specific and highly reproducible H3K27ac pattern in FGR placentas. Differentially acetylated regions were distributed throughout the whole genome as shown by Manhattan plot (Fig. 1d). Regions containing differentially acetylated peaks correspond with known H3K27ac positions derived from ENCODE databases (Fig. 1e).

### Genes annotated to within 20 kb of differentially acetylated regions

**Fig. 2:**
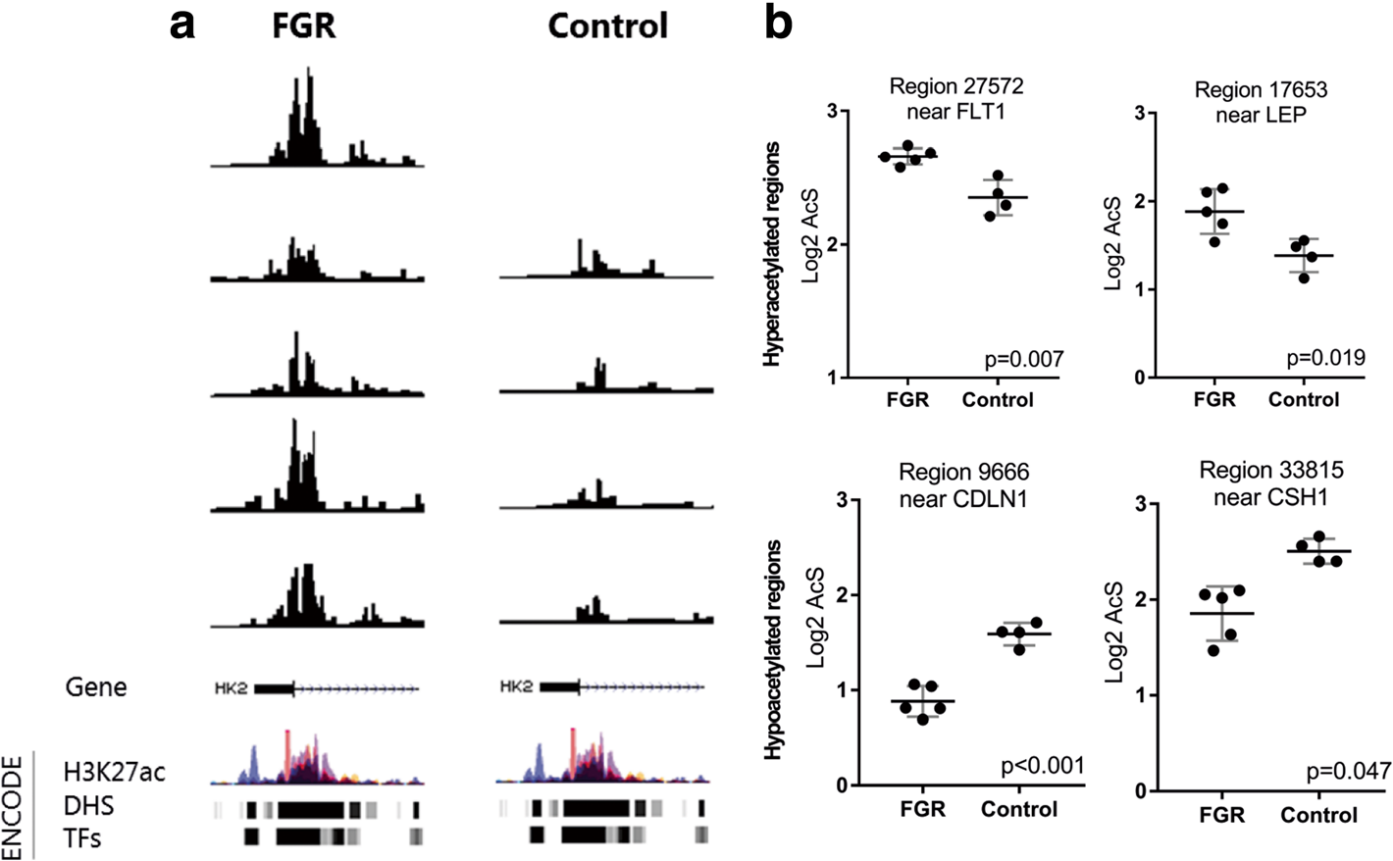
Selected regions of differentially acetylated regions in FGR placentas and related pathways. **a** A selected acetylated region near the HK2 gene in each individual sample using the USC Genome Browser showing similarity of patterns between each replicate in both groups. **b** Dot plots of four differently acetylated regions related to genes known to be involved in placental development (mean ± SD, adjusted *p* value shown)

To identify the biological relevance of differentially acetylated peaks, we annotated genes to the hyper- and hypoacetylated sites using a window of ± 20 kb from transcription start site (TSS). We annotated 368 genes corresponding to hyperacytelated sites and 313 genes associated with hypoacetylated sites (Additional file 1: Table S2). Several of these annotated genes are known to be involved in placental development. For instance, we found hyperacetylation of regions near HK2, FLT1, and LEP, previously reported to be upregulated in other placental disorders [25, 26], and hypoacetylation of regions near CH2 and CDLN1, which are involved in growth and endothelial cell-to-cell adhesions. The individual peaks of selected regions of interest are presented in Fig. 2a (near HK2) and Fig. 2b (near Flt-1, LEP, CDLN1, and CH1). These figures show that sites identified to be differentially acetylated are highly similar across each of the replicates.

### Pathway analysis of differential acetylated regions in FGR placentas

**Fig. 3:**
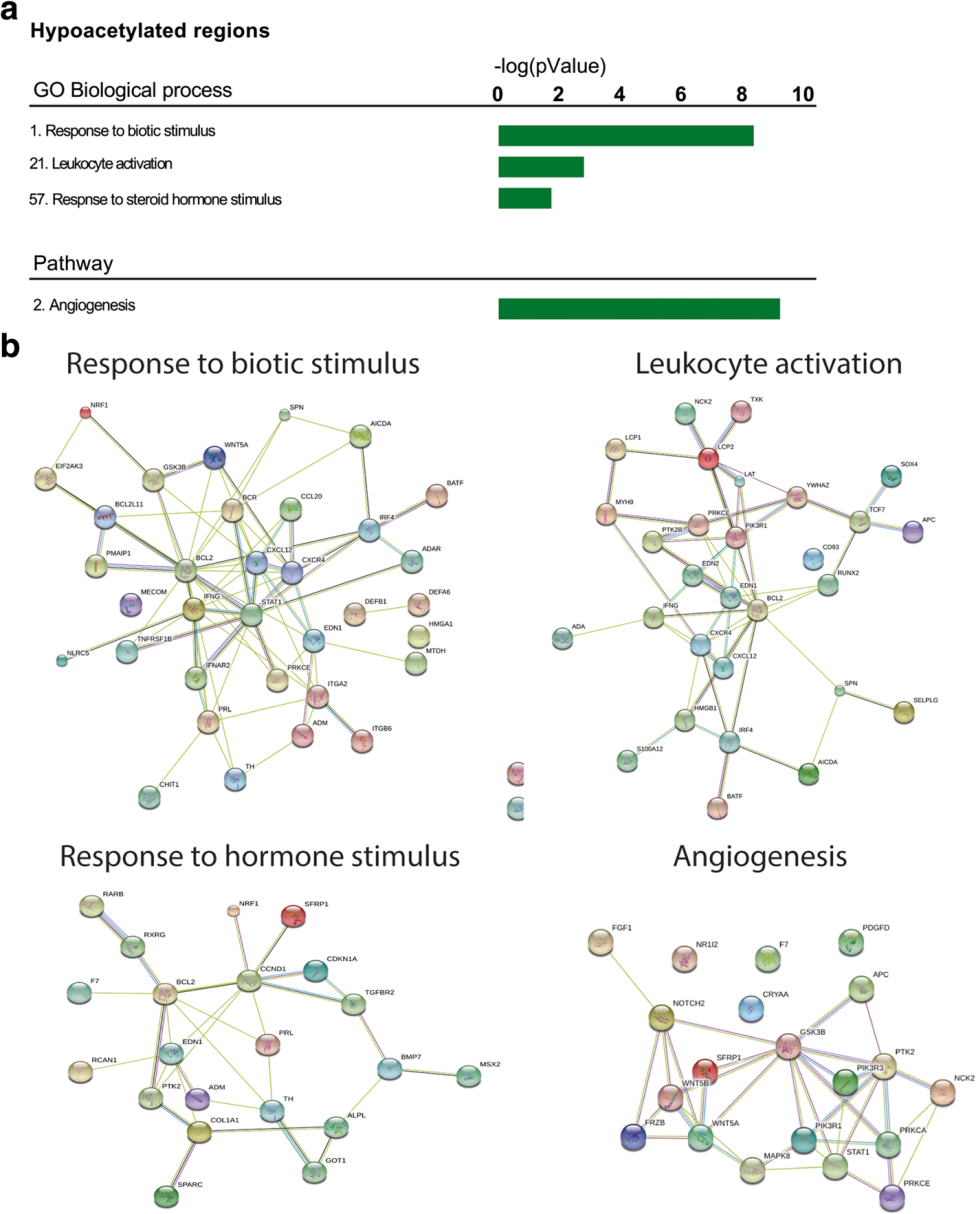
GREAT pathway analysis using differentially acetylated regions. **a** Identification of GO biological processes and pathways related to differentially acetylated regions in FGR using GREAT software. **b** Detection of interacting proteins by STRING protein database using genes annotated to biological processes and pathways related to enriched differentially acetylated regions. Only the highest confidence interactions are displayed. Disconnected nodes were removed

Next, we aimed to assign biological significance to genes annotated to differentially acetylated peaks by pathway analysis using GREAT software. The software annotated 515 genes to hyperacetylated regions in FGR. Although no enrichment for GO biological process or for pathways was identified using this approach, the annotated genes were enriched for genes that are transcriptionally regulated by HIF-1-alfa/hypoxia within the MSigDB perturbation ontology (full output supplied in Additional file 1: Table S3). Other functional pathways related to hyperacetylated regions include pathways involved in cancer and immune response. Nearby the differential hypoacetylated regions, GREAT identified 868 genes. GO biological processes and pathways related to these genes consisted of angiogenesis, response to external signals, and immune activation (Fig. 3a, b, full output supplied in Additional file 1: Table S3). Interestingly, many of these pathways are known to be disrupted in FGR, especially angiogenesis, HIF-1-alpha signaling, and the immune environment/response [27–29].

### Cross-validation of functional effects by combining H3K27ac and mRNA profiles

To cross-validate functional meaning of the differentially acetylated peaks and to examine whether hyper- or hypoacetylated state of H3K27 was also accompanied by differential gene expression, we performed RNA-seq. We identified 569 upregulated genes and 521 downregulated genes in FGR vs. control (full list supplied in Additional file 1: Table S4, heatmap and MA and V plots in Additional file 3). These gene expression profiles largely overlapped with findings from earlier gene expression studies in FGR placentas using microarrays [12–15]. This is also reflected in the biological processes and pathways associated with the up- and downregulated genes supplied in Additional file 1: Table S5. These findings confirm disruption of gene expression in placenta from FGR pregnancies involved in important processes of placental development in angiogenesis and immune modulation.

**Fig. 4:**
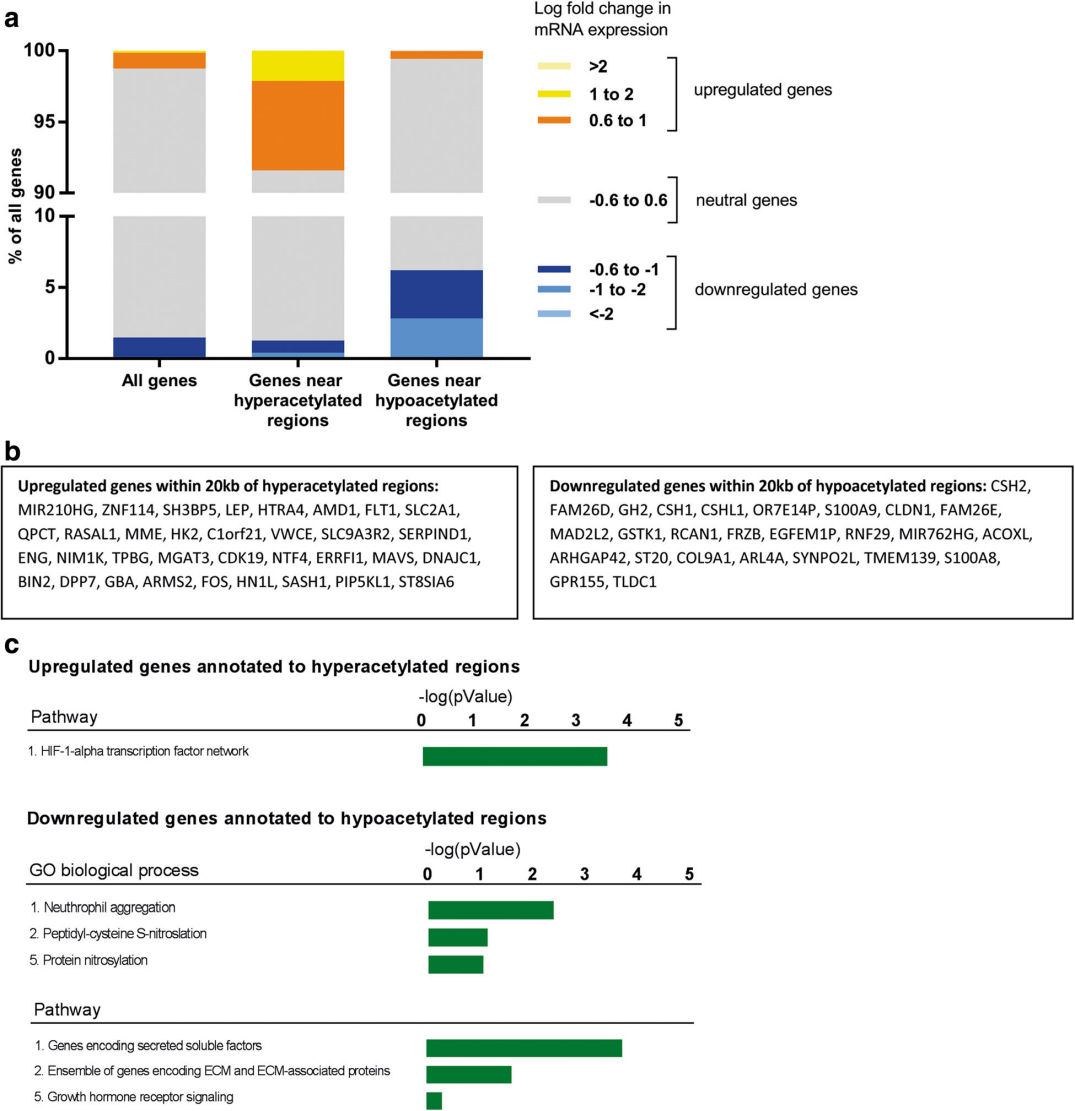
Combined analyses of CHIP-seq and RNA-seq. **a** Distribution of fold changes in gene expression near hyperacetylated and hypoacetylated regions. **b** Differentially transcribed genes detected by RNA-seq with a TSS within 20 kb of differentially acetylated regions and differentially regulated gene transcripts. **c** Identification of GO biological processes and pathways within genes overlapping in CHIP and RNA-seq regions using ToppFun

To investigate whether the direction of transcription change corresponds with differential acetylation, we investigated the distribution transcriptional direction of all genes and that of the genes annotated within 20 kb to hyperacetylated and hypoacetylated regions. Despite multiple levels/layers involved in regulation of the mRNA levels, we found a clear correlation between differential H3K27ac levels and gene expression changes (Fig. 4a). Next, we identified genes that overlapped in the gene annotation derived from differentially acetylated regions with the up- and downregulated transcripts to inspect acetylation sites that most likely influenced gene expression. We identified 34 upregulated genes in close vicinity of hyperacetylated sites and 26 downregulated genes near hypoacetylated regions (Fig. 4b). These lists contain many candidates known to be involved in the pathophysiology of FGR, such as Flt-1 and LEP, which have been described in up to one third of the studies using placentas derived from pregnancies complicated by preeclampsia [26], a placental disorder frequently associated with FGR. Other identified candidate genes included FOS, ENG (upregulated transcripts), and GH2 (downregulated transcripts).

### Identification of pathways by combining H3K27ac profiles and gene expression levels

Pathway with TOPPfun on upregulated genes near hyperacetylated sites revealed genes to be related to the HIF-1-alpha transcription factor network (Fig. 4c, Additional file 1: Table S6). In addition, the downregulated transcripts and hypoacetylated regions could be grouped as genes encoding secreted soluble factors, extracellular matrix proteins, and molecules involved in growth hormone receptor signaling (Fig. 4c, Additional file 1: Table S6).

### Transcription factor binding motif analysis

**Fig. 5:**
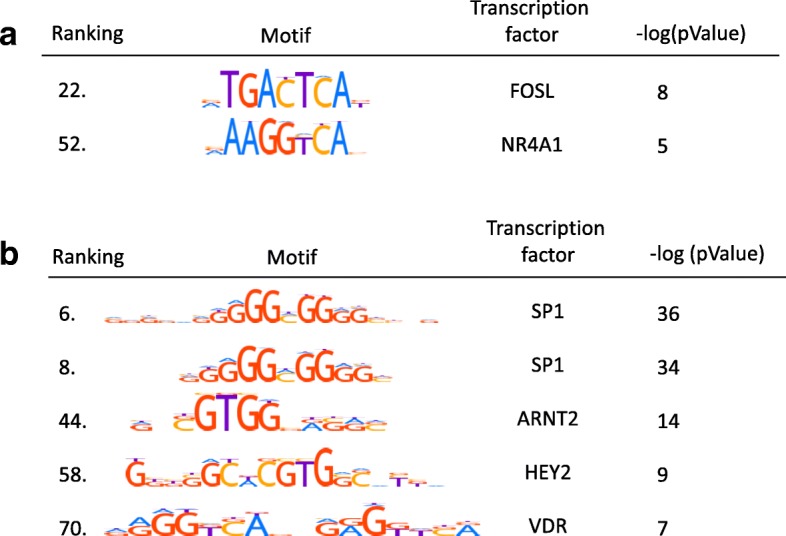
Differentially acetylated transcription factor binding motifs (TFBMs). **a** TFBMs with upregulated transcripts of their corresponding TFs. **b** TFBMs with downregulated transcripts of their corresponding TFs

To further investigate functional properties of the differential H3K27ac peaks, we tested whether the peaks contained enricanalysishment of transcription factor binding motifs (TFBM) using AME [30]. We detected 86 hyperacetylated and 55 hypoacetylated TFBM (Additional file 1: Table S7). Combining these motifs with the RNA-seq, we identified four upregulated transcription factors with enriched H3K27ac in their DNA binding domains (SP1, ARNT2, HEY2, and VDR) and two downregulated transcription factors with lower H3K27ac peaks (FOSL1, NR4A1) (Fig. 5). Of these, ARNT2 and HEY2 were previously shown to be upregulated under hypoxic conditions [31, 32]. Moreover similar changes in gene expression of VDR have been reported previously in preeclamptic placenta pathways [33], and FOSL1 was previously showed to be important for the establishment of the maternal-fetal interface [34, 35]. Collectively, these findings point towards TFs linking differentially transcribed genes with differentially acetylated regulatory chromosomal regions. Thus, transcriptional and epigenetic regulations are intricately connected in placental development.

## Discussion

In this study, we used ChIP-seq and RNA-seq to perform an in-depth analysis of DNA regions with differential chromatin activity in healthy human placentas and pathological placentas of pregnancies affected by FGR. With this combined approach, we were able to identify H3K27 acetylation as a key additional layer involved in epigenetic regulation of gene expression in placental function which in turn might be related to impaired fetal growth.

In summary, our findings confirm that, in FGR, the placenta exhibits substantial genome-wide alterations in H3K27 acetylation, with corresponding changes in transcription profiles in several regions pertinent to placental development and presumably function. The identified acetylation patterns show a clear distinction between FGR and healthy placentas, and the genes annotated to differentially acetylated regions are involved in pathways known to be affected in FGR [27–29]. We confirmed functionality of the differentially acetylated regions by showing a clear correlation between the acetylation profiles with differential gene expression. In-depth analyses of the differences in FGR placenta that revealed candidate genes and pathways that fit previously reported histopathological and protein data. For example, we found hyperacetylation and higher mRNA of the sFlt-1 region in FGR placentas, consistent with studies reporting upregulation of sFlt-1 protein in FGR placentas [36, 37]. Furthermore, sFlt-1 is known to be enriched in syncytial knots, which are also observed in FGR placenta [38]. Similarly, the leptin protein was showed to be upregulated in FGR placentas [39, 40] and GH and CSH proteins are often downregulated in FGR placentas [41, 42]. Additionally, we unmasked an interplay between differentially transcribed transcription factors with enriched TFBM within the differentially acetylated regions. Together, our data suggest an important role of H3K27ac as an additional layer/level in the regulation of placental gene expression. Our findings confirm that a combined ChIP-seq and RNA-seq approach may provide a useful approach to probe the pathophysiology of placental disease and discover novel targets to improve placental health and thus support fetal growth and development.

To date, we are not aware of any previous reports on histone modification in FGR placentas. Other groups have focused attention on epigenetic regulation of placentas in FGR involving DNA methylation. Genome-wide methylation studies in placenta of FGR suggest involvement of pathways associated with lipid metabolism, transcription, and cadherin and Wnt signaling [20–22]. Others have reported on DNA methylation of imprinted loci, since unbalanced placental expression of imprinted genes has been reported in FGR placenta [12, 43–45]. However, methylation actually fluctuates very little at these loci [45]. While DNA methylation may contribute to placental gene regulation, it can only in part explain differences in gene expression [23, 24]. Together with our results, this emphasizes the need to include other epigenetic processes to fully appreciate the complexity of mechanisms underlying placental gene expression and (primary or secondary adaptive) gene responses during (disrupted) early human development.

Mechanisms involved in differential acetylation of histones within the human placenta have not been studied. In view of the “fetal origins of adult disease” hypothesis (also known as the *Barker hypothesis*), i.e., the concept that exposure to intrauterine conditions may have long-lasting effects on fetal development, these epigenetic alterations might well be the result of changes in the intrauterine environment associated with FGR [8]. One of the intrauterine factors that might play a role in changes in epigenetic profiles in the placenta of FGR is the timing and degree of hypoxia. For example, it was showed previously that the proliferation of trophoblasts is highly dependent on the crosstalk between HIFs and histone deacetylases (HDACs) in response to hypoxia [46]. In addition, differences in histone acetylation may be the in part driven by variation in the genetic code itself, e.g., single-nucleotide polymorphisms, that shape chromatin architecture and thereby disrupt normal placental development [47, 48]. Considering potential intervention, it is of great interest to study the contribution of both environmental and intrinsic factors in more detail.

Our study has several strengths. First, our data provide new and comprehensive information on epigenetic regulation of placental gene expression in FGR. Our data suggest that H3K27ac profiles have a role in genome-wide regulation of gene expression in FGR placenta and point towards disruptions in important pathways involved in placental development. Histone modifications represent mid-long-term effects [49] and are likely more stable and independent of sample moment compared to RNA, and by combining CHIP-seq data with RNA-seq data, we were able to relate epigenetic marks to functional gene expression patterns, confirming relevance of identified pathways. Another strength is our careful collection and detailed phenotyping of FGR placentas during Cesarean sections, excluding those women who have labored. Thus, we avoided confounding effects of temporal changes associated with parturition [50].

There are some limitations in our approach that also need to be addressed. First, we could not avoid the limitation of the use of nearest gene approach for functional annotation. Here, we used a 20-kb window to annotate genes which, although quite wide, still allowed identification of specific pathways and genes that corresponded with previous literature. With regard to the collected material, there were unavoidable difference in gestational ages in material from FGR and control due to the severity of the selected FGR cases. It is possible that gestational age in itself has an effect on H3K27ac and gene expression as shown in early mouse placenta [51] and for other epigenetic marks such as methylation as shown in human placenta [21]. While gestational age might be a confounder, the strongest determinant in our cohort is likely to be the disease, given that all placentas were from third trimester pregnancies while majority of gene expression changes occur earlier in development [52] and the confirmed disease-specific pathways. In addition, largest differences in epigenetics in the third trimester are mainly induced by parturition [50], which we ruled out by collecting samples from C-sections only.

With this study, we show that genome-wide H3K27ac profiles might be useful to better understand pathophysiology of FGR. Therefore, we would recommend future studies to confirm the identified acetylation targets in a larger population as this might be helpful to address association of the differentially acetylated regions with different subtypes of FGR, gestational-age specific effects on acetylation within the third trimester of healthy and FGR pregnancy, and, most importantly, to explore cause-effect relationships. The latter might be very valuable as the plasticity of histone marks form an attractive strategy for intervention, especially since no therapy is currently available to improve placental function. In addition, it will be valuable to study the combination of other epigenetic marks, such as DNA methylation and other histone marks, to better discriminate promoters from enhancers (H3K4me3, H3K4me1) or repressed chromatin (H3K27me3) [53]. Moreover, since placental samples represent an average state across different cellular compartments with trophoblast being the predominant cell type [54], newer techniques looking at subpopulations of cells might help to unravel cell-specific deregulated pathways [55].

## Conclusions

We demonstrate involvement of H3K27ac in key regions related to the placental function and placental pathology associating with impaired fetal growth. Our findings underscore that an approach using combined CHIP-seq and RNA-seq analyses facilitates discovery of novel genes involved in FGR, and identification of molecular pathways and processes associated with the placental function and early (disrupted) human growth and development. Future studies on unraveling the role of H3K27ac in FGR pregnancies and placental-fetal development may help to identify novel targets for therapy of this currently incurable disease.

## Methods

### Study design and sample collection

Placenta biopsies were collected immediately after birth from women with a FGR pregnancy and women with uncomplicated pregnancy. Each placenta was sampled at four random locations. For this study, we only included women undergoing a primary Cesarean section. We defined FGR as an estimated fetal weight <p3 and only included cases with pulsatility index of the uterine artery >p97.5 since we were particularly interested in cases with FGR based on placenta insufficiency. Controls were included when planned for Cesarean section because of either breech presentation or history of Caesarian section. Additional file 4 provides an overview of characteristics from the pregnancies of which the placenta were derived. The material was snap-frozen directly after sampling and stored at − 80 °C. Before further processing, all four biopsies per placenta were pooled and ground into powder using mortar and pestle cooled with liquid nitrogen.

### ChIP-sequencing for H3K27ac occupancy

The powdered samples, two scoops diluted in 700 μl sPBS, were used for chromatin isolation using the MAGnify™ Chromatin Immunoprecipitation System kit (Life Technologies, Thermo Fisher Scientific, Carlsbad, CA) according to manufacturer’s instructions. In brief, the tissue was crosslinked with 1% formaldehyde and the crosslinking was stopped by adding 1.25 M glycine. Cells were lysed using the kit-provided lysis buffer, and nuclei were sonicated using Covaris microTUBE (duty cycle 5%, intensity 2, 200 cycles per burst, 60 s cycle time, 10 cycles). We aimed for DNA fragments of 200–2000 bp long. We continued with one fifth of the volume, and the sheared chromatin was diluted and then incubated with 1 μl anti-H3K27ac antibody (ab4729, Abcam) pre-coupled to magnetic beads for 2 h at 4 °C. Beads were extensively washed, and crosslinking was reversed by the kit-provided reverse crosslinking buffer with proteinase K. DNA was purified using ChIP DNA Clean & Concentrator kit (Zymo Research). Libraries were prepared using the NEXTflex™ Rapid DNA Sequencing Kit (Bioo Scientific). Samples were PCR-amplified and checked for the proper size range and for the absence of adaptor dimers on a 2% agarose gel, and barcoded libraries were sequenced 75 bp single-end on Illumina NextSeq500 sequencer (Utrecht Sequencing Facility).

### Mapping of ChIP-sequencing reads

Sequencing reads were mapped against the reference genome (hg19 assembly, GRCh37) using BWA package (mem –t 7 –c 100 –M –R) [56]. Multiple reads mapping to the same location and strand have been collapsed to single reads, and only uniquely placed reads were used for peak/region calling. Regions were called using Cisgenome 2.0 ( –e 150 -maxgap 200 –minlen 200) [57]. Subsequently, to obtain a common reference, region coordinates from all FGR and control samples were stretched to at least 2000 bp and collapsed into a single common list. Overlapping regions were merged based on their outmost coordinates. Only the autosomal regions supported by at least two independent datasets were further analyzed. Sequencing reads from each ChIP-seq library were overlapped with the common region list, to set the H3K27ac occupancy for every region-sample pair.

### ChIP data analysis

Regions with differential H3K27ac occupancy between FGR samples and controls were identified using DESeq2 (*p* < 0.05 by Wald test) [58] and are referred to as “differentially acetylated regions.” Hierarchical clustering based on differentially acetylated regions was performed with quantile-normalized, log2-transformed, and median-centered read counts per common region. To avoid log2 transformation of zero values, one read was added to each region. A Manhattan plot was created representing the distribution of regions detected to be differentially acetylated in FRG vs. controls across autosomal chromosomes. The unsupervised PCA analysis was performed for the top 500 most variable regions using DESeq2. Based on the initial analysis, we excluded one control sample because of being an extreme outlier in the PCA analysis and continued the analysis with *n* = 5 FGR and *n* = 4 control samples.

To assign biological meaning to differentially acetylated peaks, we used three approaches. First, we annotated genes to the differentially acetylated sites in silico using a window of ± 20 kb from transcription start site (TSS). Second, GREAT software (Stanford) was used to assign biological meaning to a set of non-coding genomic regions by analyzing the annotations of genes flanking differentially acetylated regions [59]. GREAT incorporates annotations from 20 ontologies and accounts for the length of gene regulatory domains. ToppFun was used for gene list enrichment analysis and candidate gene prioritization based on functional annotations and protein interaction networks (accessed in March 2017). In addition, we identified whether the differentially acetylated regions contain enrichment of specific transcription factor binding domains by overlapping the differentially hypo- and hyperacetylated regions in the FGR vs. control group to placenta DNAse hypersensitivity site (DHS) datasets obtained from the ENCODE database (ENCFF203HVV, ENCFF249GZW, ENCFF919NRH). The genomic sequence of overlapping DHS was repeat masked, and the enrichment of TFBM was calculated against a random set of non-overlapping DHS sequences using the Analysis Motif of Enrichment (AME tool) of the MEME Suite with default settings using human (HOCOMOCO v9) motif database.

### RNA sequencing

Total RNA was extracted from placental powder using RNeasy® (Qiagen, Hilden, Germany) according to the manufacturer’s instructions by which all RNA molecules longer than 200 nucleotides are purified. Polyadenylated mRNA fraction was isolated using Poly(A) Beads (NEXTflex, San Jose, CA), and sequencing libraries were constructed using the Rapid Directional RNA-seq kit (NEXTflex, San Jose, CA). Libraries were sequenced on the Nextseq500 platform (Illumina, San Diego, CA), producing single-end reads of 75 bp (Utrecht Sequencing Facility). Reads were aligned to the human reference genome GRCh37 using STAR version 2.4.2a. Picard’s AddOrReplaceReadGroups (v1.98) was used to add read groups to the BAM files, which were sorted with Sambamba v0.4.5, and transcript abundances were quantified with HTSeq-count version 0.6.1p1 using the union mode. Subsequently, reads per kilobase million reads sequenced (RPKMs) were calculated with edgeR’s RPKM function.

### RNA-seq data analysis

Differentially expressed genes were identified using the DESeq2 package with standard settings. Genes with padj < 0.05 were considered as differentially expressed. Again, ToppFun was used for gene list enrichment analysis and candidate gene prioritization (accessed in March 207). The list of up- and downregulated genes were tested separately using probability density function *p* value calculation, FDR B&H correction, *p* value cutoff 0.05.

### ChIP-seq and RNA-seq overlap analyses

To validate functional consequences of differentially acetylated regions, we overlapped the genes annotated to differentially hyperacetylated regions with upregulated genes derived from the mRNA-seq and genes annotated to differentially hypoacetylated regions with downregulated genes derived from the mRNA-seq. Functional pathway analysis with TOPPFUN were performed on both sets of overlapping genes. In addition, we searched the up- and downregulated genes for transcription factors (TF) with enriched TFBM within the differentially acetylated regions.

### Statistical analysis

Data are shown as mean ± SD or median (range) and analyzed with Student’s *t* test or *χ*^2^ test.

## Additional files

Additional file 1: **Table S1**. Differentially acetylated regions in FGR compared to controls. **Table S2.** Genes annotated to hyper- and hypoacetylated sites in placenta of FGR using a 20-kb window. **Table S3**. Functional pathways related to hypo- and hyperacetylated in placenta of FGR identified by GREAT. **Table S4.** Up- and downregulated gene transcripts in placenta of FGR compared to controls. **Table S5.** RNA-seq pathways TOPPfunn. **Table S6.** Functional pathways related to overlapping genes in CHIP-seq and RNA-seq. **Table S7.** AME output: differentially acetylated TFBM’s in placenta of FGR vs. controls and functional pathways related to differentially acetylated TFBM (XLSX 694 kb)

Additional file 2: Heatmap, MA plot, and V plot CHIP-seq. (DOCX 175 kb)

Additional file 3: Heatmap, MA plot, and V plot RNA-seq. (DOCX 120 kb)

Additional file 4: Pregnancy characteristics of the placenta samples. (DOCX 15 kb)

## References

[1] Ernst J, Kheradpour P, Mikkelsen TS, Shoresh N, Ward LD, Epstein CB (2011). Mapping and analysis of chromatin state dynamics in nine human cell types. Nature.

[2] Creyghton MP, Cheng AW, Welstead GG, Kooistra T, Carey BW, Steine EJ (2010). Histone H3K27ac separates active from poised enhancers and predicts developmental state. Proc Natl Acad Sci U S A.

[3] Mirabella AC, Foster BM, Bartke T (2016). Chromatin deregulation in disease. Chromosoma.

[4] Hnisz D, Abraham BJ, Lee TI, Lau A, Saint-Andre V, Sigova AA (2013). Super-enhancers in the control of cell identity and disease. Cell.

[5] Romo A, Carceller R, Tobajas J (2009). Intrauterine growth retardation (IUGR): epidemiology and etiology. Pediatr Endocrinol Rev.

[6] Bernstein IM, Horbar JD, Badger GJ, Ohlsson A, Golan A (2000). Morbidity and mortality among very-low-birth-weight neonates with intrauterine growth restriction. The Vermont Oxford Network. Am J Obstet Gynecol.

[7] Barker DJ, Osmond C, Golding J, Kuh D, Wadsworth ME (1989). Growth in utero, blood pressure in childhood and adult life, and mortality from cardiovascular disease. BMJ.

[8] Kermack AJ, Van Rijn BB, Houghton FD, Calder PC, Cameron IT, Macklon NS (2015). The “developmental origins” hypothesis: relevance to the obstetrician and gynecologist. J Dev Orig Health Dis.

[9] Chaddha V, Viero S, Huppertz B, Kingdom J (2004). Developmental biology of the placenta and the origins of placental insufficiency. Semin Fetal Neonatal Med.

[10] Veerbeek JHW, Brouwers L, Koster MPH, Koenen SV, van Vliet EOG, Nikkels PGJ (2016). Spiral artery remodeling and maternal cardiovascular risk: the spiral artery remodeling (SPAR) study. J Hypertens.

[11] Veerbeek JHW, Nikkels PGJ, Torrance HL, Gravesteijn J, Post Uiterweer ED, Derks JB (2014). Placental pathology in early intrauterine growth restriction associated with maternal hypertension. Placenta.

[12] McMinn J, Wei M, Schupf N, Cusmai J, Johnson EB, Smith AC (2006). Unbalanced placental expression of imprinted genes in human intrauterine growth restriction. Placenta.

[13] McCarthy C, Cotter FE, McElwaine S, Twomey A, Mooney EE, Ryan F (2007). Altered gene expression patterns in intrauterine growth restriction: potential role of hypoxia. Am J Obstet Gynecol.

[14] Struwe E, Berzl G, Schild R, Blessing H, Drexel L, Hauck B (2010). Microarray analysis of placental tissue in intrauterine growth restriction. Clin Endocrinol.

[15] Madeleneau D, Buffat C, Mondon F, Grimault H, Rigourd V, Tsatsaris V (2015). Transcriptomic analysis of human placenta in intrauterine growth restriction. Pediatr Res.

[16] Nelissen ECM, van Montfoort APA, Dumoulin JCM, Evers JLH (2011). Epigenetics and the placenta. Hum Reprod Update.

[17] Bianco-Miotto T, Mayne BT, Buckberry S, Breen J, Rodriguez Lopez CM, Roberts CT (2016). Recent progress towards understanding the role of DNA methylation in human placental development. Reproduction.

[18] Lewis RM, Cleal JK, Hanson MA (2012). Review: placenta, evolution and lifelong health. Placenta.

[19] Schroeder DI, Blair JD, Lott P, Yu HOK, Hong D, Crary F (2013). The human placenta methylome. Proc Natl Acad Sci U S A.

[20] Roifman M, Choufani S, Turinsky AL, Drewlo S, Keating S, Brudno M (2016). Genome-wide placental DNA methylation analysis of severely growth-discordant monochorionic twins reveals novel epigenetic targets for intrauterine growth restriction. Clin Epigenetics.

[21] Hillman SL, Finer S, Smart MC, Mathews C, Lowe R, Rakyan VK (2015). Novel DNA methylation profiles associated with key gene regulation and transcription pathways in blood and placenta of growth-restricted neonates. Epigenetics.

[22] Lambertini L, Lee T-L, Chan W-Y, Lee M-J, Diplas A, Wetmur J (2011). Differential methylation of imprinted genes in growth-restricted placentas. Reprod Sci.

[23] Joo JE, Hiden U, Lassance L, Gordon L, Martino DJ, Desoye G (2013). Variable promoter methylation contributes to differential expression of key genes in human placenta-derived venous and arterial endothelial cells. BMC Genomics.

[24] Lopez-Abad M, Iglesias-Platas I, Monk D (2016). Epigenetic characterization of CDKN1C in placenta samples from non-syndromic intrauterine growth restriction. Front Genet.

[25] Kaartokallio T, Cervera A, Kyllonen A, Laivuori K, Kere J, Laivuori H (2015). Gene expression profiling of pre-eclamptic placentae by RNA sequencing. Sci Rep.

[26] Kleinrouweler CE, van Uitert M, Moerland PD, Ris-Stalpers C, van der Post JAM, Afink GB (2013). Differentially expressed genes in the pre-eclamptic placenta: a systematic review and meta-analysis. PLoS One.

[27] Gourvas V, Dalpa E, Konstantinidou A, Vrachnis N, Spandidos DA, Sifakis S (2012). Angiogenic factors in placentas from pregnancies complicated by fetal growth restriction (review). Mol Med Rep.

[28] Kimura C, Watanabe K, Iwasaki A, Mori T, Matsushita H, Shinohara K (2013). The severity of hypoxic changes and oxidative DNA damage in the placenta of early-onset preeclamptic women and fetal growth restriction. J Matern Fetal Neonatal Med.

[29] Prins JR, Faas MM, Melgert BN, Huitema S, Timmer a HMN (2012). Altered expression of immune-associated genes in first-trimester human decidua of pregnancies later complicated with hypertension or foetal growth restriction. Placenta.

[30] McLeay RC, Bailey TL (2010). Motif enrichment analysis: a unified framework and an evaluation on ChIP data. BMC Bioinformatics.

[31] Mandl M, Depping R (2014). Hypoxia-inducible aryl hydrocarbon receptor nuclear translocator (ARNT) (HIF-1beta): is it a rare exception?. Mol Med.

[32] Diez H, Fischer A, Winkler A, Hu C-J, Hatzopoulos AK, Breier G (2007). Hypoxia-mediated activation of Dll4-Notch-Hey2 signaling in endothelial progenitor cells and adoption of arterial cell fate. Exp Cell Res.

[33] Ma R, Gu Y, Zhao S, Sun J, Groome LJ, Wang Y (2012). Expressions of vitamin D metabolic components VDBP, CYP2R1, CYP27B1, CYP24A1, and VDR in placentas from normal and preeclamptic pregnancies. Am J Physiol Endocrinol Metab.

[34] Kent LN, Rumi MAK, Kubota K, Lee D-S, Soares MJ (2011). FOSL1 is integral to establishing the maternal-fetal interface. Mol Cell Biol.

[35] Soares MJ, Chakraborty D, Renaud SJ, Kubota K, Bu P, Konno T (2012). Regulatory pathways controlling the endovascular invasive trophoblast cell lineage. J Reprod Dev.

[36] Nevo O, Many A, Xu J, Kingdom J, Piccoli E, Zamudio S (2008). Placental expression of soluble FMS-like tyrosine kinase 1 is increased in singletons and twin pregnancies with intrauterine growth restriction. J Clin Endocrinol Metab.

[37] Hoeller A, Ehrlich L, Golic M, Herse F, Perschel FH, Siwetz M (2017). Placental expression of sFlt-1 and PlGF in early preeclampsia vs. early IUGR vs. age-matched healthy pregnancies. Hypertens pregnancy.

[38] Rajakumar A, Cerdeira AS, Rana S, Zsengeller Z, Edmunds L, Jeyabalan A (2012). Transcriptionally active syncytial aggregates in the maternal circulation may contribute to circulating soluble FMS-like tyrosine kinase 1 in preeclampsia. Hypertens (Dallas, Tex 1979).

[39] Schrey S, Kingdom J, Baczyk D, Fitzgerald B, Keating S, Ryan G (2013). Leptin is differentially expressed and epigenetically regulated across monochorionic twin placenta with discordant fetal growth. Mol Hum Reprod.

[40] Li RHW, Poon SCS, Yu MY, Wong YF (2004). Expression of placental leptin and leptin receptors in preeclampsia. Int J Gynecol Pathol.

[41] Velegrakis A, Sfakiotaki M, Sifakis S (2017). Human placental growth hormone in normal and abnormal fetal growth. Biomed reports.

[42] Mannik J, Vaas P, Rull K, Teesalu P, Laan M (2012). Differential placental expression profile of human growth hormone/chorionic somatomammotropin genes in pregnancies with pre-eclampsia and gestational diabetes mellitus. Mol Cell Endocrinol.

[43] Diplas AI, Lambertini L, Lee M-J, Sperling R, Lee YL, Wetmur J (2009). Differential expression of imprinted genes in normal and IUGR human placentas. Epigenetics.

[44] Iglesias-Platas I, Martin-Trujillo A, Petazzi P, Guillaumet-Adkins A, Esteller M, Monk D (2014). Altered expression of the imprinted transcription factor PLAGL1 deregulates a network of genes in the human IUGR placenta. Hum Mol Genet.

[45] Camprubi C, Iglesias-Platas I, Martin-Trujillo A, Salvador-Alarcon C, Rodriguez MA, Barredo DR (2013). Stability of genomic imprinting and gestational-age dynamic methylation in complicated pregnancies conceived following assisted reproductive technologies. Biol Reprod.

[46] Maltepe E, Krampitz GW, Okazaki KM, Red-Horse K, Mak W, Simon MC (2005). Hypoxia-inducible factor-dependent histone deacetylase activity determines stem cell fate in the placenta. Development.

[47] Handy DE, Castro R, Loscalzo J (2011). Epigenetic modifications: basic mechanisms and role in cardiovascular disease. Circulation.

[48] Miguel-Escalada I, Pasquali L, Ferrer J (2015). Transcriptional enhancers: functional insights and role in human disease. Curr Opin Genet Dev.

[49] Turner BM (2007). Defining an epigenetic code. Nat Cell Biol.

[50] Lee KJ, Shim SH, Kang KM, Kang JH, Park DY, Kim SH (2010). Global gene expression changes induced in the human placenta during labor. Placenta.

[51] Tuteja G, Chung T, Bejerano G (2016). Changes in the enhancer landscape during early placental development uncover a trophoblast invasion gene-enhancer network. Placenta.

[52] Uusküla L, Männik J, Rull K, Minajeva A, Kõks S, Vaas P (2012). Mid-gestational gene expression profile in placenta and link to pregnancy complications. PLoS One.

[53] Karlic R, Chung H-R, Lasserre J, Vlahovicek K, Vingron M (2010). Histone modification levels are predictive for gene expression. Proc Natl Acad Sci U S A.

[54] Bonet B, Brunzell JD, Gown AM, Knopp RH (1992). Metabolism of very-low-density lipoprotein triglyceride by human placental cells: the role of lipoprotein lipase. Metabolism.

[55] Gormley M, Ona K, Kapidzic M, Garrido-Gomez T, Zdravkovic T, Fisher SJ (2017). Preeclampsia: novel insights from global RNA profiling of trophoblast subpopulations. Am J Obstet Gynecol.

[56] Li H, Durbin R (2009). Fast and accurate short read alignment with Burrows-Wheeler transform. Bioinformatics.

[57] Ji H, Jiang H, Ma W, Johnson DS, Myers RM, Wong WH (2008). An integrated software system for analyzing ChIP-chip and ChIP-seq data. Nat Biotechnol.

[58] Love MI, Huber W, Anders S (2014). Moderated estimation of fold change and dispersion for RNA-seq data with DESeq2. Genome Biol.

[59] McLean CY, Bristor D, Hiller M, Clarke SL, Schaar BT, Lowe CB (2010). GREAT improves functional interpretation of cis-regulatory regions. Nat Biotechnol.

