# Supplementary material for: H3K27 acetylation and gene expression analysis reveals differences in placental chromatin activity in fetal growth restriction
Source: Clin Epigenetics. 2018 Jun 26;10:85. doi: 10.1186/s13148-018-0508-x (PMC6020235; doi:10.1186/s13148-018-0508-x)
Supplement: Supplementary file 2 — Heatmap, MA plot, and V plot CHIP-seq. (DOCX 175 kb) [file 13148_2018_508_MOESM2_ESM.docx]

**
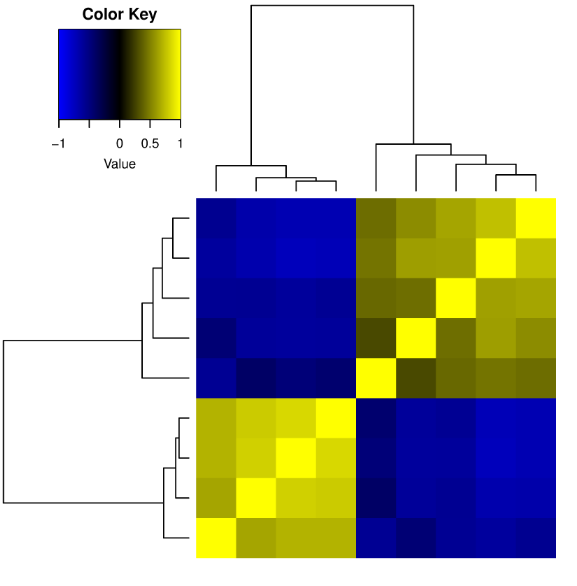
Additional file 2 Heatmap using differentially acetylated regions (A), MA plot (B) and V plot (C).**

**A**

**Control FGR**

**
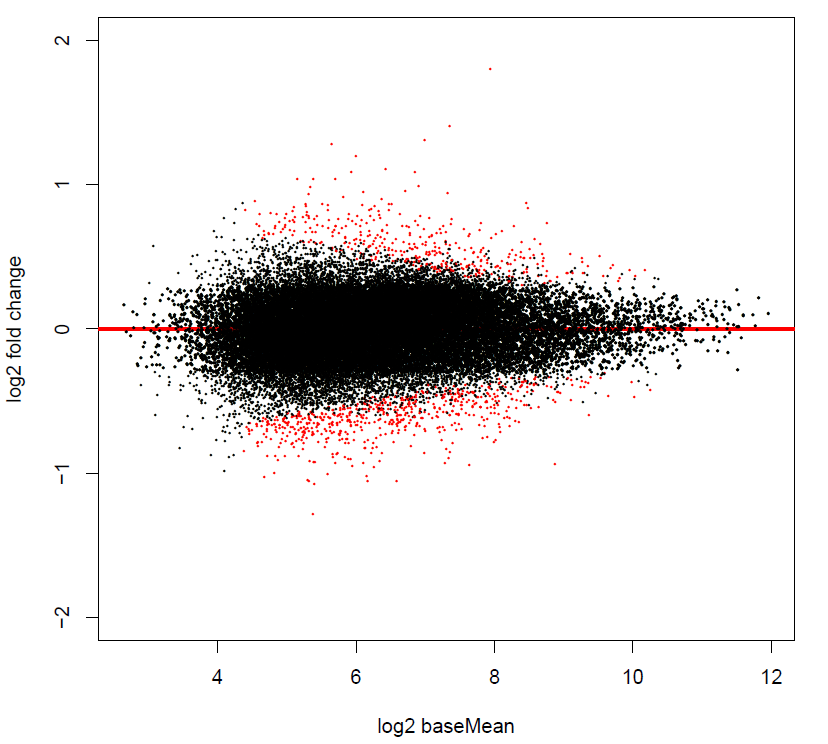
**
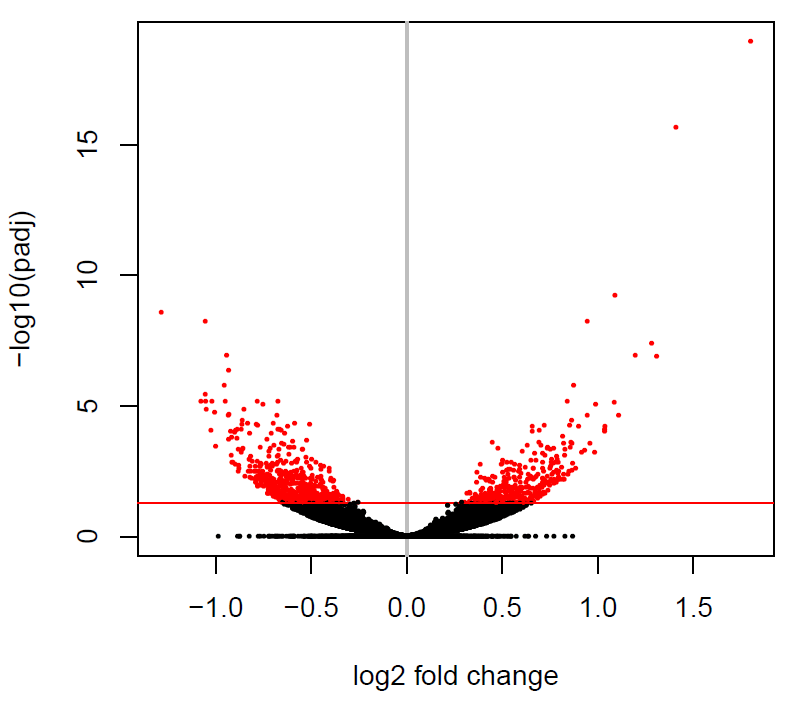
**B C**
