# Supplementary material for: H3K27 acetylation and gene expression analysis reveals differences in placental chromatin activity in fetal growth restriction
Source: Clin Epigenetics. 2018 Jun 26;10:85. doi: 10.1186/s13148-018-0508-x (PMC6020235; doi:10.1186/s13148-018-0508-x)
Supplement: Supplementary file 3 — Heatmap, MA plot, and V plot RNA-seq. (DOCX 120 kb) [file 13148_2018_508_MOESM3_ESM.docx]

**Additional file 3 Heatmap using differentially expressed genes (A), MA plot (B) and V plot (C).**

**
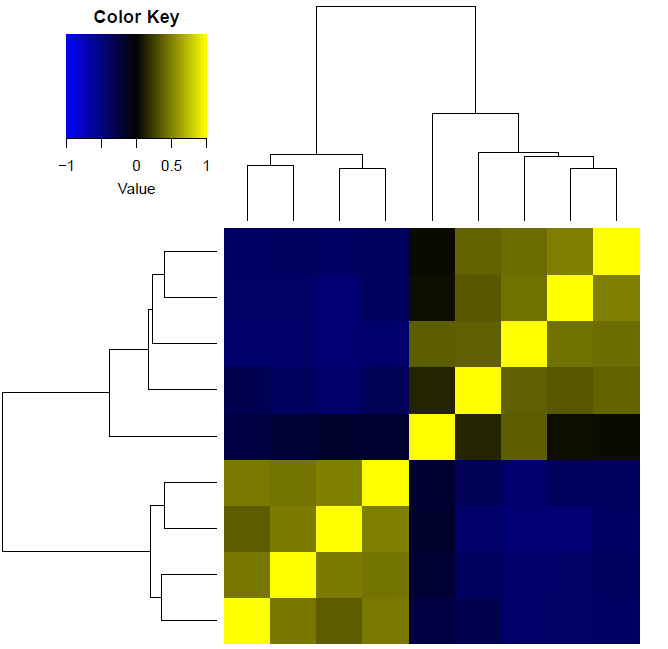
A**

**Control FGR**

**
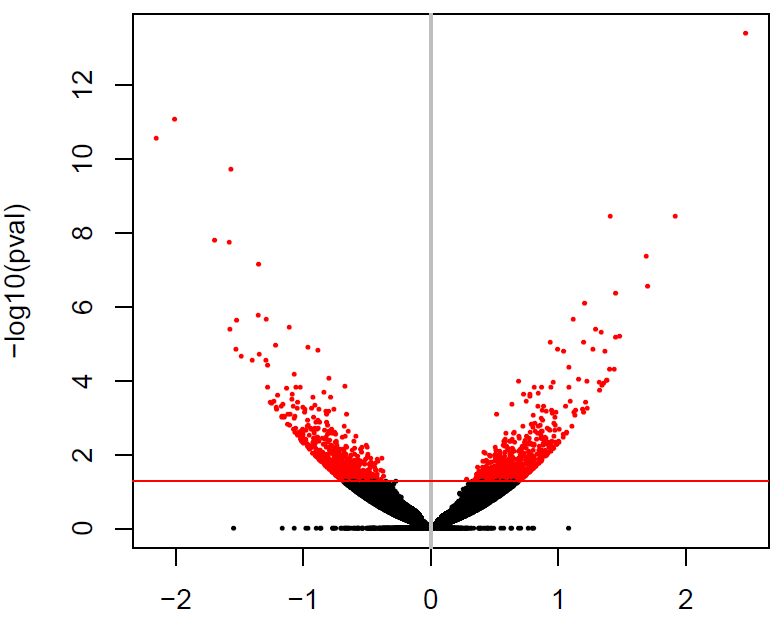

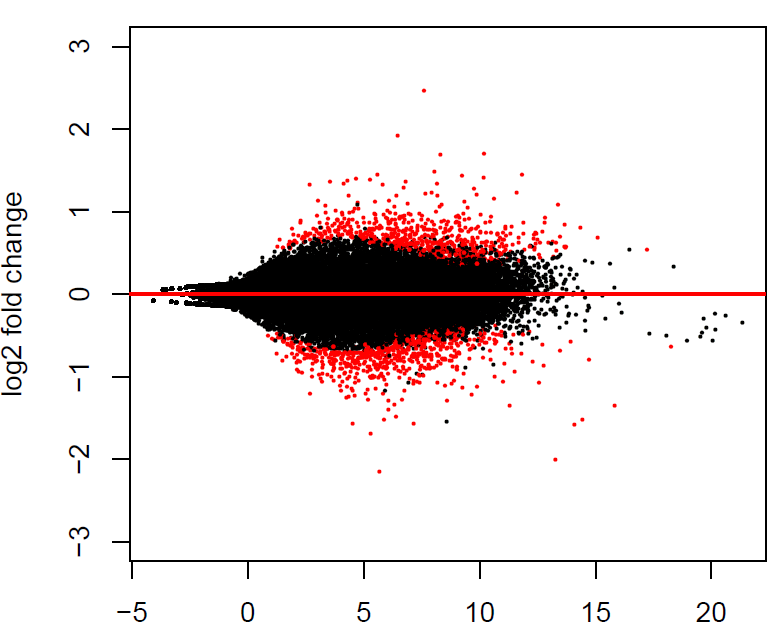
B C**
