# Supplementary material for: H3K27 acetylation and gene expression analysis reveals differences in placental chromatin activity in fetal growth restriction
Source: Clin Epigenetics. 2018 Jun 26;10:85. doi: 10.1186/s13148-018-0508-x (PMC6020235; doi:10.1186/s13148-018-0508-x)
Supplement: Supplementary file 4 — Pregnancy characteristics of the placenta samples. (DOCX 15 kb) [file 13148_2018_508_MOESM4_ESM.docx]

**Additional file 1 Pregnancy characteristics of the placenta samples**

|  | **IUGR (n= 5)** | **Control (n=4)** | *P-value* |
| --- | --- | --- | --- |
| **Maternal age (years; mean ± SD)** | 30 ± 7 | 34 ± 7 | 0.40 |
| **Parity**  Nullipara (n (%))  Multipara (n (%)) | 5 (100)  0 (0) | 2 (50)  2 (50) | 0.17 |
| **Gestational age at delivery (weeks+days; median (range))** | 30+4 (28+1-33+2) | 39+3 (39+0-40+5) | **0.01** |
| **Birth weight**  **(grams; median (range))** | 1085 (725-1445) | 3493 (3115-3950) | **0.01** |
| **Sex of child**  Male (n (%))  Female (n (%)) | 1 (20)  4 (80) | 3 (75)  1 (25) | 0.10 |
| **Flow umbilical artery** Normal (n (%))  Null/reversed (n (%)) | 1 (20) 4 (80) | 4 (100)  0 (0) | **0.02** |
| **Hypertensive pregnancy complications**  None (n (%))  Preeclampsia (n (%) | 1 (20)  4 (80) | 4 (100)  0 (0) | **0.02** |
| **Reason for CS**  CIF (n (%)  History of CS (n (%))  Maternal indication (n (%))  Fetal distress (n (%)) | 0 (0)  0 (0)  2 (40)  3 (60) | 3 (75)  1 (25)  0 (0)  0 (0) | **0.03** |
